# Real-Time Terrain Recognition for Quadruped Robots Using Proprioceptive Sensors and Temporal Convolutional Networks

**DOI:** 10.3390/s26134050

**Published:** 2026-06-25

**Authors:** Tzu-Hsiu Chang, Minyechil Alehegn Tefera, Jun-Ming Cheng, Tsung-Ming Fang, Chin-Sheng Chen, Chia-Jen Lin, Peng-Chun Peng, Chao-Ching Ho, Tzu-Hsuan Tsai, Cherng-Yuh Su, Shih-Hao Chang, Pai-Yen Chen, Hsiang-Wei Ho, Ching-Yuan Chang

**Affiliations:** 1 Department of Mechanical Engineering, National Taipei University of Technology, Taipei 10608, Taiwan; t107669006@ntut.org.tw (T.-H.C.); minyechil.alehegn@ntut.edu.tw (M.A.T.); t112568049@ntut.edu.tw (J.-M.C.); t113408069@ntut.org.tw (T.-M.F.); hochao@mail.ntut.edu.tw (C.-C.H.); cysu@ntut.edu.tw (C.-Y.S.); 2 Graduate Institute of Automation Technology, National Taipei University of Technology, Taipei 10608, Taiwan; saint@ntut.edu.tw; 3 Department of Electrical Engineering, National Yunlin University of Science and Technology, Douliou 64002, Taiwan; cjlin@yuntech.edu.tw; 4 Department of Electro-Optical Engineering, National Taipei University of Technology, Taipei 10608, Taiwan; pcpeng@ntut.edu.tw; 5 Department of Materials and Mineral Resources Engineering, National Taipei University of Technology, Taipei 10608, Taiwan; tzhtsai@ntut.edu.tw; 6 Department of Computer Science and Information Engineering, National Taipei University of Technology, Taipei 10608, Taiwan; sh.chang@ntut.edu.tw; 7 Department of Electrical and Computer Engineering, University of Illinois Chicago, Chicago, IL 60607, USA; pychen@uic.edu; 8 Department of Industrial Technology, Ministry of Economic Affairs, R.O.C., Taipei 100210, Taiwan; hwho@moea.gov.tw; 9 Department of Mechanical Engineering, Asia Eastern University of Science and Technology, New Taipei City 220303, Taiwan

**Keywords:** quadruped robots, proprioceptive sensors, sensor fusion, real-time terrain classification, deep learning, temporal convolutional networks (TCNs), slip detection

## Abstract

In this article, we propose a novel real-time terrain recognition and slip estimation method for quadruped robots using proprioceptive sensors and temporal convolutional networks (TCNs). As quadruped robots are increasingly deployed in complex environments, accurate terrain understanding is crucial. External sensors can be affected by lighting variations, occlusion, reflective surfaces, and others. To overcome these challenges, we propose a proprioceptive sensing-based complementary perception module with a TCN, enabling reliable real-time terrain recognition while reducing dependence on external perception. The TCN model effectively captures temporal dependencies in sensor signals, enabling precise and robust detection. The framework is validated through extensive real-world experiments and deployed on an embedded edge computing platform for real-time operation. Results show that the proposed TCN method achieves 98.8% recognition accuracy, outperforming the baseline models compared in this study. In addition, this study analyzes how locomotion speed and environmental conditions affect slip in quadruped robots. These findings confirm that quadruped robots can not only recognize terrain types but also detect surface states, enabling safer and more adaptive locomotion. Therefore, the proposed system is a cost-effective, robust, and low-latency solution for real-time terrain recognition, providing a strong foundation for future deployment across more diverse terrains.

## 1. Introduction

In recent years, robots have become a transformative force in modern society, playing a crucial role in enhancing productivity, safety, and quality of life across various domains. With their ability to integrate perception, decision-making, and execution, they have become a key driving force in industrial automation. Moreover, they enable flexible and intelligent manufacturing in smart factories [1] and in precision agriculture [2], and they enhance production efficiency and product quality [3], autonomous navigation [4], and others [5,6,7,8]. In [1], researchers studied human–robot perception technologies in industrial environments and highlighted their role in improving operational safety and efficiency. In addition, Botta et al. [2] summarized the application of robotic systems in precision agriculture for monitoring and field operations. Heyer [3] discussed future industrial robotic applications and their impact on manufacturing productivity. Meanwhile, Wijayathunga et al. [8] studied navigation and scene-understanding techniques for autonomous ground robots in unstructured environments. These studies collectively demonstrate the application of robots, growing importance of sensing, perception, and control technologies in modern robotic systems. However, wheeled and tracked robots cannot navigate uneven terrain, stairs, or obstacles, making them unsuitable for real-world, unstructured environments. Compared with wheeled and tracked robots, quadrupedal robots offer relatively better mobility on uneven terrain [9,10]. However, their performance still remains insufficient to meet the requirements of demanding applications in unstructured environments or challenging terrain, including industrial inspection, outdoor exploration, military operations, disaster search and rescue, and other real-world tasks. Furthermore, most existing approaches rely on assumptions of static environments or complete environmental knowledge, which rarely hold in practical deployments characterized by dynamic and partially observable conditions, and they are often validated only in simulation rather than on real robots [7,11]. To address these problems, we propose and experimentally demonstrate a robust framework tested in real-world environments, showcasing its effectiveness and adaptability under dynamic environments.

Previous terrain perception approaches rely on external sensors such as cameras and LiDAR, which are often affected by lighting variations, occlusion, and increased system cost and complexity [7,8,12]. The authors in [12] proposed a perceptive locomotion framework for quadrupedal robots with a main output of control policy that integrates exteroceptive and proprioceptive sensing. However, external sensors may become unreliable in challenging conditions such as lighting variation, occlusion, reflective surfaces, or other field-deployment uncertainties, and their use increases system complexity and cost. Therefore, complementary sensing strategies are required to improve robustness in real-world deployments. To address this problem, we propose a proprioceptive sensor and TCN-based terrain classification and slip estimation method for quadruped robots, which leverages internal sensing data, including inertial measurement units (IMUs) and actuator feedback signals, to identify ground types without relying on external sensors. Moreover, proprioceptive sensors are compact, cost-effective, and robust, making them particularly suitable for field deployment. In addition, future challenges for field robots focus on achieving greater autonomy for long-duration missions. Accurate terrain understanding is essential to meet this goal. By optimizing control strategies and planning energy-efficient paths, robots can operate safely, avoid getting stuck, and complete their missions more effectively. Therefore, we propose an AI-based approach that addresses these challenges and enables reliable terrain classification in real-world environments.

Different methods have been proposed in recent years to classify and perceive terrain for robotics systems [11,13,14,15,16]. In [8], scholars studied depth-sensing and vision-based perception systems for autonomous ground robots operating in unstructured environments. Although these sensors enable effective scene understanding and navigation, they remain susceptible to environmental conditions, sensing limitations, and computational complexity. Also, as stated in [11], researchers investigated terrain classification for quadruped robots using machine learning techniques based on locomotion data; however, their approach was evaluated on a limited set of terrain conditions. Kim et al. [13] compared a back-propagation neural network (BPNN) and support vector machine (SVM) for terrain classification in legged robots, reporting average recognition rates of 78.6% for BPNN and 78.75% for SVM when PCA-based features were used. Although these results demonstrated the feasibility of data-driven terrain classification, the study was constrained by relatively simple classifiers, low accuracy, hand-crafted feature extraction, and a simplified one-leg experimental platform. Li et al. [14] reviewed tactile sensing technologies for robotic perception and manipulation but highlighted challenges related to sensor integration, durability, and scalability. Moreover, Guastella and Muscato [16] surveyed learning-based perception and navigation methods for ground vehicles in complex environments and noted that many existing approaches rely heavily on exteroceptive sensors and computationally intensive processing. Although these studies have achieved promising results, many depend on external sensing systems that are susceptible to lighting variations, occlusions, weather conditions, and increased computational complexity. In addition, terrain maps generated online are frequently imperfect due to factors such as sensor noise, obstacles, reflections, and others [17]. Moreover, traditional machine learning methods rely on handcrafted features and often fail to capture temporal dependencies [13,18]. Consequently, these traditional methods and classification algorithms remain difficult to apply effectively in real-world robotic applications. Moreover, deep learning models such as LSTMs and CNNs have shown considerable promise for time-series analysis. However, they often encounter challenges in capturing long-term dependencies and maintaining computational efficiency [19]. To overcome these limitations, we propose TCNs for real-time terrain classification in quadruped robots. TCNs can effectively model sequential data over time, enabling them to learn dynamic patterns in robot–terrain interaction signals and achieve more accurate and robust terrain recognition [19,20,21,22]. Moreover, TCN is computationally efficient and more stable, making it well-suited for real-time operation in dynamic and unstructured environments. Thus, TCNs provide an effective solution for real-time terrain classification in quadruped robots.

**Figure 1:**
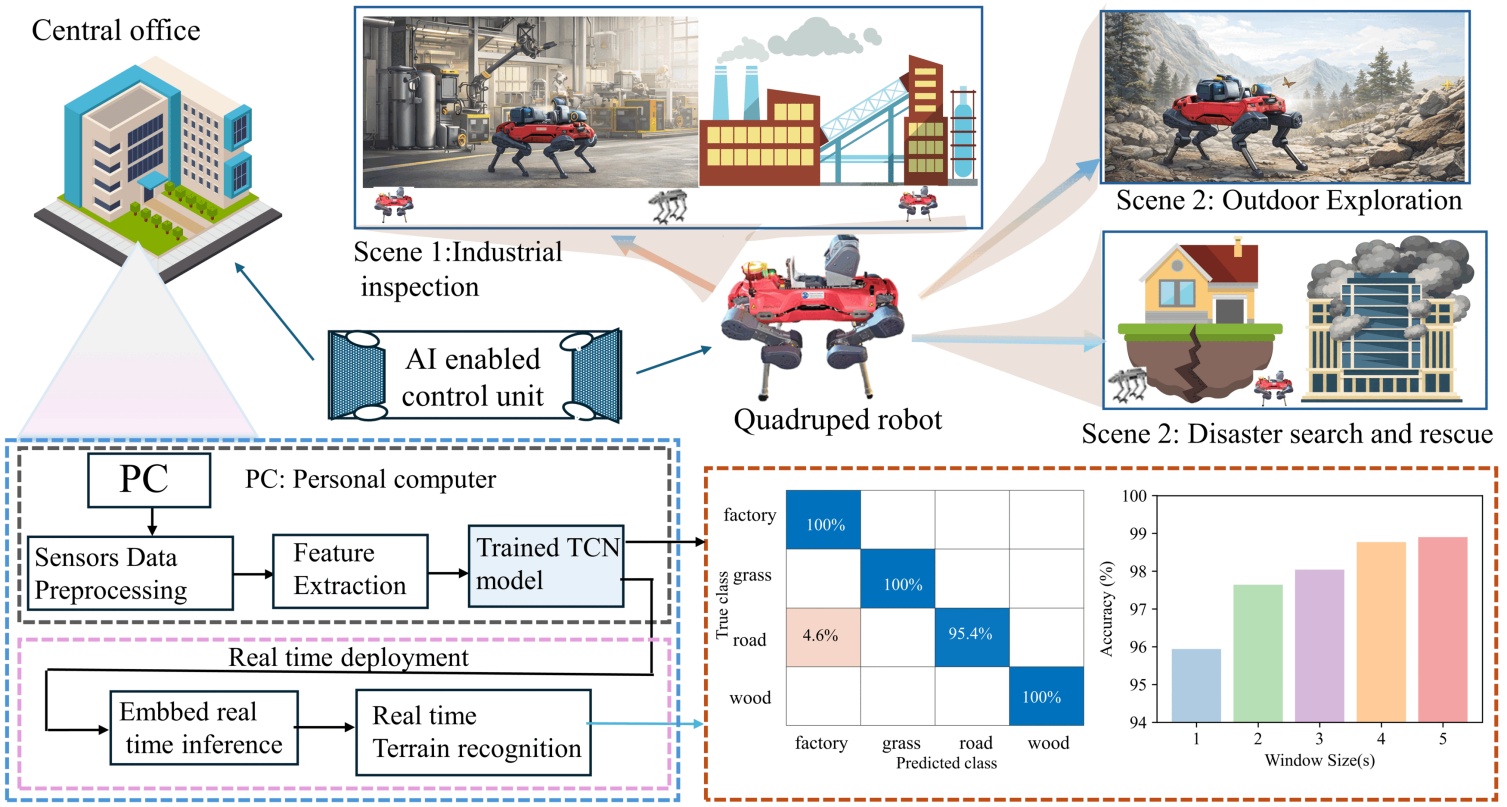
Conceptual overview of the proposed system.

Figure 1 shows an overview of the proposed terrain-aware control framework for quadruped robots. Terrain understanding plays a critical role in enhancing the mobility, stability, and autonomy of quadruped robots operating in complex environments. The figure presents a centralized AI-enabled control pipeline, where raw proprioceptive sensor data are first preprocessed and transformed through feature extraction, followed by training using a Temporal Convolutional Network (TCN). The trained model is deployed on an embedded platform to enable real-time inference, supporting continuous terrain classification and slip-aware locomotion control. The framework is validated across multiple application scenarios, including industrial inspection, outdoor exploration, and disaster search and rescue, demonstrating strong adaptability and robustness. Performance results, illustrated through a confusion matrix and accuracy analysis, indicate high classification accuracy and highlight the effectiveness of incorporating temporal context.

In this paper, a real-time terrain classification and slip estimation approach for quadruped robots based on an internal sensor system is proposed. Instead of relying on external sensors, which are often affected by lighting variations and occlusion and are costly, our method uses proprioceptive sensor fusion data. The torque spectrum reflects the dynamic interaction between the robot’s legs and the ground, providing valuable information about terrain characteristics. By analyzing these spectral features through TCNs, the proposed system can accurately and robustly classify various terrain types in real time. This approach enhances the adaptability and autonomy of quadruped robots, enabling them to navigate unstructured and unpredictable environments more effectively. The main contributions of this work are listed as follows:

- The proposed method relies on proprioceptive sensors to provide a complementary terrain-state recognition module without requiring additional external perception hardware. Rather than replacing cameras or LiDAR, the method reduces dependence on exteroceptive sensing by enabling robust contact-based terrain classification and slip estimation, when external perception is degraded by lighting variations, occlusion, reflective surfaces, or environmental uncertainty. In addition, the proposed method offers low-cost internal sensing, utilizes a TCN to capture temporal robot–terrain interactions, supports embedded real-time deployment, and improves operational reliability in environments where external perception becomes unreliable.
- Terrain features are extracted from the frequency-domain characteristics of joint torque signals, offering a new perspective for terrain understanding.
- The proposed TCN-based approach effectively learns temporal dependencies in the torque spectrum, achieving accurate and robust terrain classification, outperforming other models presented in this paper.
- A slip-aware sensing module is incorporated to assess terrain-specific slip risk under different speeds and environmental conditions, supporting safer and more adaptive locomotion.
- Real-time validation on an embedded quadruped platform demonstrates the practicality, low latency, and robustness of the proposed sensing framework in real-world environments.

The remainder of this article is organized as follows. Section 2 presents the experimental setup, followed by Section 3, which details the research methodology. Section 4 discusses the obtained results and their implications. Finally, Section 5 concludes the paper.

## 2. Experimental Setup

**Figure 2:**
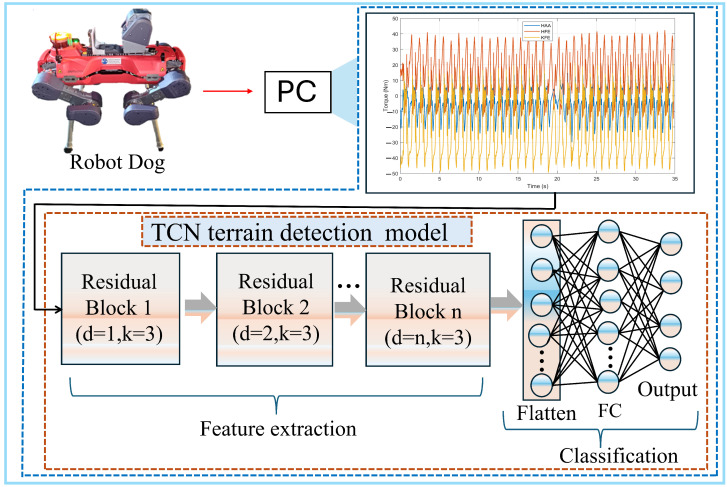
Experimental setup of the proposed real-time terrain recognition system for quadruped robots (TCN = temporal convolutional networks; FC = Fully connected).

Figure 2 shows the experimental setup of the proposed real-time terrain classification and slip estimation system for quadruped robots. Experiments were performed using the ANYmal D quadruped platform, equipped with onboard sensing, control, and computing modules. The embedded system was built on a NVIDIA Jetson Orin NX (16 GB) running the robot operating system (ROS) for real-time communication and data management. The robot was manually controlled using the graphical user interface (GUI) provided by the ANYmal software suite (release 25.06). Through this interface, the operator commanded standard locomotion actions, including forward and backward walking and left and right turns. For each terrain type, such as epoxy floor, interlocking bricks, grass, and hardwood decking boards, the robot executed these motion sequences continuously, and approximately 45 min of data were collected per terrain. Each leg joint of the quadruped robot was equipped with torque sensors, while an inertial measurement unit (IMU) was mounted on the body to record global motion. Torque sensors measured the rotational forces exerted at each actuator joint. The torque readings represent the interaction forces between the robot’s feet and the ground. The IMU captured three-axis linear acceleration and angular velocity, describing the robot’s body motion, vibration, and orientation during locomotion. The accelerometer detected oscillations due to surface roughness, and the gyroscope recorded angular variations during turning and gait transitions. Together, the torque and IMU signals provided a comprehensive representation of leg–ground interaction and body dynamics, serving as multi-modal input features for terrain classification. The data were then synchronized and saved in ROS bag format for post-processing. Finally, the measured and recorded data are transferred from ROS to the PC for further machine learning tasks.

## 3. Proposed Methodologies

### 3.1. Data Collection and Annotations

**Figure 3:**
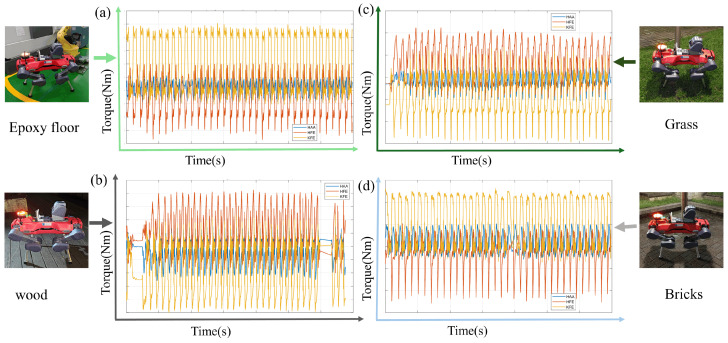
Sample torque response of the ANYmal D quadruped robot while walking on four different surfaces: (**a**) epoxy floor, (**b**) wood, (**c**) grass, and (**d**) bricks.

The experimental configuration used to collect the sensor data is illustrated in Figure 2. In addition, Figure 3 shows the torque response of the ANYmal quadruped robot when walking on four different surfaces: (a) epoxy floor, (b) wood, (c) grass, and (d) bricks. The hip abduction adduction (HAA), hip flexion extension (HFE), and knee flexion extension (KFE) joint torques exhibit terrain-specific patterns as shown in Figure 3a–d. The data collection process was conducted in three experimental scenarios to capture diverse locomotion patterns and improve the generalization of the terrain classification model. In scenario A, the robot performed straight-line walking maneuvers across four terrains—epoxy floor, interlocking bricks, grass, and hardwood decking boards—with approximately 35 s of data collected per terrain. In scenario B, the robot executed forward walking combined with left and right rotations for 30 min per terrain to increase motion diversity and enhance the robustness of the dataset. In scenario C, the robot performed backward walking and rotational movements for 30 min per terrain to simulate complex maneuvers and further enrich the dataset. In total, 120 min of synchronized sensor data were recorded at a sampling rate of 200 Hz, with each second containing 200 samples of joint torque, torque derivative (torque/dt), joint acceleration, and IMU signals from all 12 joints and the body sensor recorded using the Jetson Orin NX through ROS. The subscribed topics—/sensors/imu and /anymal_low_level_controller/actuator_readings—were saved in ROS bag format and segmented into multiple five-minute recordings for each terrain. Subsequently, the recorded ROS bag files were converted into CSV format. All data were normalized using Z-scores to eliminate scale differences among features. The normalization can be computed as follows:

(1) $$z_{i}=\frac{x_{i}-\mu_{i}}{\sigma_{i}}$$

where $\mu_{i}$ and $\sigma_{i}$ represent the mean and standard deviation of the *i*th feature, respectively.

Even though the sensor data were configured to sample at 200 Hz, the recorded ROS bag files exhibit slight temporal irregularities due to system factors. The asynchronous nature of multisensor acquisition, ROS message scheduling, and Jetson Orin NX computational load may cause slight jitter in timestamps and sampling intervals during real-time logging. To address this practical limitation and ensure robustness in frequency-domain feature extraction, the Lomb–Scargle periodogram (LSP) was employed. Unlike conventional Fourier-based methods, LSP does not require strictly uniform sampling and is therefore well-suited for handling real-world robotic datasets where minor timing inconsistencies may exist. Therefore, a LSP is adopted to ensure robust spectral analysis under weakly non-uniform sampling conditions. For a torque signal $\tau_{i}=\tau \left(t_{i}\right)$, the Lomb–Scargle power spectrum at angular frequency ω is defined as follows:

(2) $$\begin{array}{cc} P_{x}\left(\omega \right)= & \frac{1}{2\sigma^{2}}[\frac{{\left(\sum_{i=1}^{N}\left(\tau_{i}-\bar{\tau }\right)\cos\omega \left(t_{i}-\hat{\tau }\right)\right)}^{2}}{\sum_{i=1}^{N}\cos^{2}\omega \left(t_{i}-\hat{\tau }\right)}\\ \; & +\frac{{\left(\sum_{i=1}^{N}\left(\tau_{i}-\bar{\tau }\right)\sin\omega \left(t_{i}-\hat{\tau }\right)\right)}^{2}}{\sum_{i=1}^{N}\sin^{2}\omega \left(t_{i}-\hat{\tau }\right)}] \end{array}$$

where *N* is the number of samples, $\bar{\tau }$ and $\sigma^{2}$ denote the sample mean and variance of the signal, respectively, and $\hat{\tau }$ is a time-shift parameter that ensures phase invariance. The parameter $\hat{\tau }$ is given by

(3) $$\tan\left(2\omega \hat{\tau }\right)=\frac{\sum_{i=1}^{N}\sin\left(2\omega t_{i}\right)}{\sum_{i=1}^{N}\cos\left(2\omega t_{i}\right)}.$$

Equation (2) was used to calculate the spectral power of the torque signal at each angular frequency. This power value represents the vibration strength of the robot–terrain interaction at a specific frequency. Equation (3) defines the phase-shift parameter, which reduces the influence of phase variation and irregular sampling. In this study, the resulting Lomb–Scargle spectral features are extracted from the joint torque signal within each gait window and then used as frequency-domain descriptors for terrain classification. This allows the system to extract stable vibration-related features from non-uniformly sampled proprioceptive data and improves the robustness of terrain recognition under embedded real-time operating conditions.

### 3.2. Proposed Temporal Convolutional Networks (TCNs)

Deep learning models have demonstrated remarkable performance in modeling sequential data due to their ability to capture complex temporal dependencies [23]. However, these models often suffer from limitations such as difficulty in parallelization, vanishing or exploding gradients, high computational cost for long sequences, and high detection errors [24,25]. Moreover, in this study, we propose a TCN-based approach to address these challenges by leveraging causal and dilated convolutions, which enable long-range temporal dependencies while maintaining efficient parallel computation. Unlike RNNs, TCNs provide a stable gradient flow through residual connections, enabling deeper architectures without the risk of vanishing gradients [26]. Furthermore, it exhibits superior flexibility in handling sequences of varying lengths, faster training convergence, and lower memory requirements, making it particularly advantageous for time-series forecasting, signal processing, and other sequential learning tasks [27]. These characteristics establish TCNs as a robust alternative to traditional methods, combining high accuracy with computational efficiency. Therefore, for our system, we propose a TCN to fully exploit these advantages and achieve superior performance in sequential data modeling. Figure 1 at the bottom shows the TCN architecture, while Algorithm 1 presents the training process of the proposed model for real-time terrain classification in quadruped robots. First, the proposed TCN model takes the sensor spectrum as its primary input, capturing the temporal-frequency characteristics of the robot’s locomotion. In TCN, a sequence of residual blocks with increasing dilation factors (*d* = 1, 2, …, *n*) and kernel size (*k* = 3) enables multi-scale temporal feature extraction while maintaining efficient computation. To capture spectral features spanning multiple gait cycles, TCN uses dilated convolution. The principle of dilated convolution is defined as follows:

(4) $$F\left(S\right)=\left(x*df\right)\left(s\right)=\sum_{i=0}^{k-1}f\left(i\right)\;x_{s-d\cdot i}$$

where *f* denotes convolution filter, *k* kernel size, *d* dilation factor, and s−d·i convolution on past states only. In addition, the effective receptive field is calculated as follows:

(5) $$\mathrm{ERF}=1+\sum_{i=1}^{L}\left(k-1\right)d_{i}$$

where *L* is the total number of layers. By increasing $d_{i}$, the TCN can observe longer temporal histories without significantly increasing the number of parameters [26]. To build a deep architecture and prevent degradation of terrain features during propagation, TCN incorporates residual connections and layer normalization, and it is computed as follows:

(6) y=ReLU(x+F(x))

where *x* denotes the identity mapping and F(x) represents the residual mapping, composed of dilated convolution layers, dropout, and weight normalization, which is responsible for learning fine-grained terrain features. Then, the extracted high-level representations are flattened and passed through fully connected (FC) layers to perform the final terrain-type classification, allowing the robot to adapt dynamically to varying ground conditions in real time. In addition, optimizers are crucial in machine learning, as they adjust the model’s parameters to minimize loss during training [28,29,30]. The Adam optimizer reduces loss during training faster than other optimizers, such as the adaptive gradient algorithm (Adagrad), adaptive delta algorithm (Adadelta), and stochastic gradient descent algorithm (SGD) [31,32]. Thus, the Adam optimizer is applied to ensure convergence of the proposed TCN method. Table 1 shows the details of the proposed TCN model hyperparameters. The proposed model calculates the loss using the categorical cross-entropy function. The loss can be computed as follows:

(7) $${\mathrm{CE}}_{i}=-\sum_{j=1}^{C}y_{i,j}\log\left({\hat{y}}_{i,j}\right)$$

where *C* is the total number of classes, $y_{i,j}$ is the ground-truth label of the *i*-th sample for class *j*, ${\hat{y}}_{i,j}$ is the predicted probability of the *i*-th sample belonging to class *j*, and ${\mathrm{CE}}_{i}$ is the categorical cross-entropy loss for the *i*-th sample.
**Algorithm 1** Proposed TCN model**Input:** Proprioceptive sensor dataset.**Output:** Trained TCN model parameters $\theta^{*}$.// *Data preprocessing***1:** Convert the raw sensor data into the required file format.**2:** Normalize the sensor data using z-score normalization.// *Dataset splitting***3:** Split the normalized sensor data into two independent subsets: Training set $D_{\mathrm{train}}=\{S_{\mathrm{train}}^{i},$$Y_{\mathrm{train}}^{i}\}$, Testing set $D_{\mathrm{test}}=\left\{S_{\mathrm{test}}^{i},Y_{\mathrm{test}}^{i}\right\}$.// *Data segmentation***4:** Segment $D_{\mathrm{train}}$ and $D_{\mathrm{test}}$ separately into overlapping time windows of length *T*.**5:** Generate labeled samples from each segmented window for terrain classification.// *Model construction***6:** Initialize the TCN model architecture.**7:** Set hyperparameters, including kernel size, dropout rate, batch size, learning rate, and optimizer.// *Training process***8: for each** epoch ≤E **do****9:**     **for each** batch ≤B **do****10:**       Select a mini-batch $\left(X_{i},y_{i}\right)$ from $D_{\mathrm{train}}$.**11:**       Feed $X_{i}$ into the TCN model and obtain predicted label ${\hat{y}}_{i}$.**12:**       Compute cross-entropy loss:$$\mathrm{loss}=-\sum_{j=1}^{C}y_{i,j}\log\left({\hat{y}}_{i,j}\right)$$**13:**       Update network parameters θ using back-propagation.**14:**     **end for****15: end for**// *Testing phase***16:** Use the trained TCN model to predict terrain labels for $D_{\mathrm{test}}$.**17:** Evaluate classification performance using testing dataset.

### 3.3. Trajectory Reconstruction and Slip Displacement Detection

**Figure 4:**
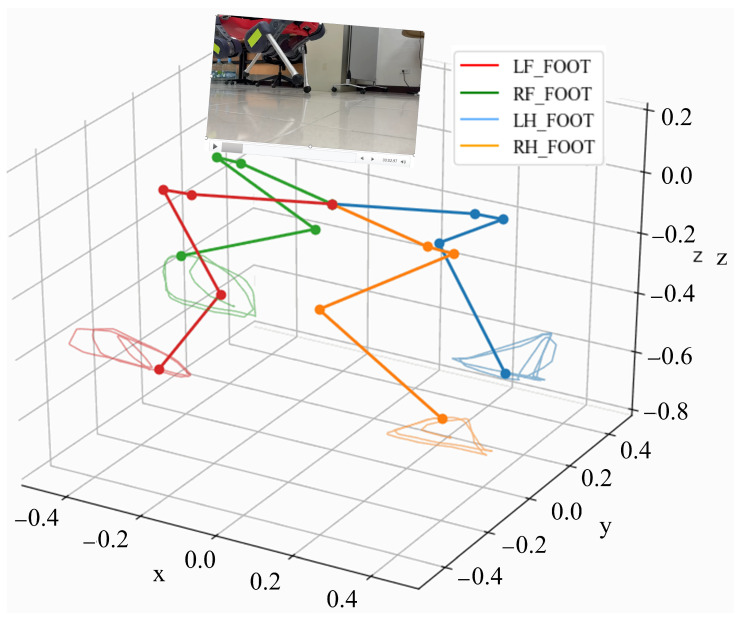
Schematic of the real-time reconstructed robot foot-end motion trajectories.

**Figure 5:**
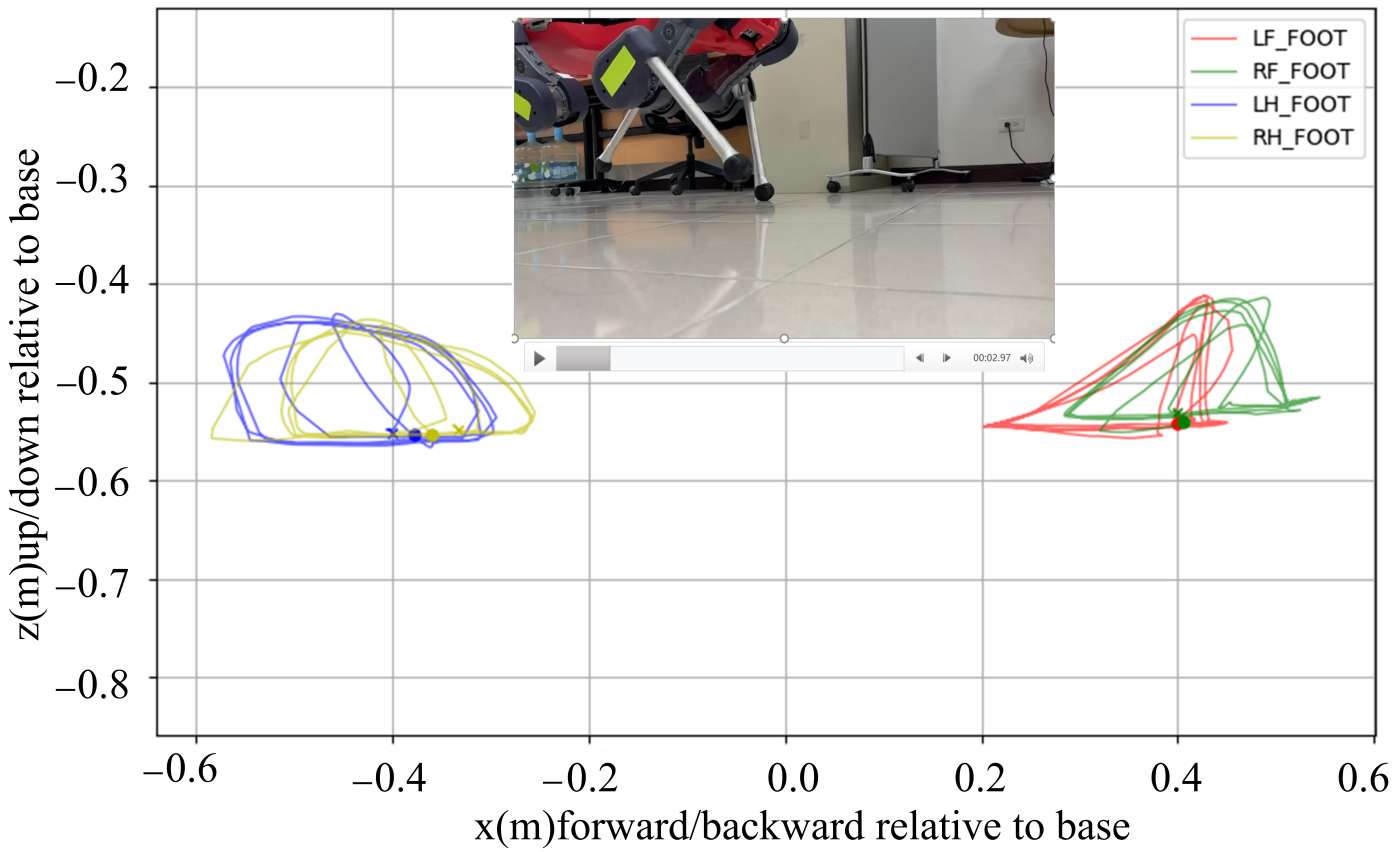
Schematic of the robot foot-end trajectory reconstructed in the XZ plane.

In this study, we propose a real-time foot-end displacement estimation framework based on a URDF-driven forward kinematics model and a slip displacement integration algorithm. The URDF model is used to compute forward kinematics, enabling the real-time, accurate reconstruction of the foot-end position relative to the robot base. Figure 4 and Figure 5 present the reconstructed foot-end trajectories.

#### Knee Flexion–Extension (KFE)-Based Impact-Triggered Slip Detection Algorithm

To improve robustness in detecting ground contact and slip initiation, a KFE-based impact-trigger mechanism is introduced. The algorithm exploits the high sensitivity of the knee flexion–extension (KFE) joint to impact dynamics during foot–ground interaction.

The system continuously monitors the KFE joint angular acceleration $Acc_{KFE}$ and the foot vertical velocity jump $v_{z,jump}$. An impact event is triggered when both conditions are satisfied:

(8) $$\left|{Acc}_{KFE}\right|>60.0\;{\mathrm{rad}/s}^{2}\;\mathrm{AND}\;v_{z,jump}>0.2\;m/s$$

This condition ensures that only high-confidence ground contact events activate the slip detection module, thereby reducing false detections caused by normal gait dynamics.

Once an impact event is detected at time $t_{start}$, a slip observation window is activated until $t_{end}$. The end of the window is defined as the moment when the foot transitions from forward motion to backward relative motion. During this process: Swing phase: $v_{x,base}>0$; Stance phase: $v_{x,base}<0$. If $v_{x,base}$ remains positive after impact, it indicates that the foot has not fully stabilized on the ground, implying ongoing slip behavior.

Within the slip window, the slip displacement is computed by numerically integrating the foot’s horizontal velocity:

(9) $$D_{slip}=\sum_{t=t_{start}}^{t_{end}}v_{x,base}\left(t\right)\cdot \Delta t$$

This formulation captures the cumulative micro-slippage during the transient contact phase, providing a quantitative measure of slip severity.

### 3.4. Embedded System Integration and Real-Time Deployment

**Figure 6:**
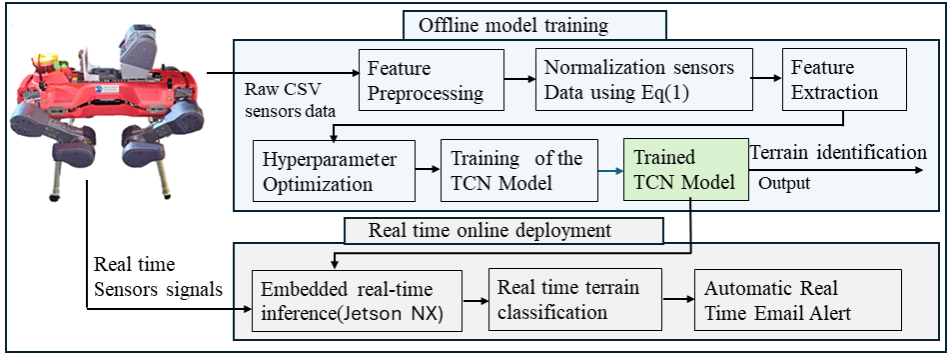
Schematic of the proposed terrain recognition system with real-time embedded inference.

The proposed system has been thoroughly tested in real-world environments, demonstrating robust performance, low latency, reliable terrain perception, and practical applicability for quadruped robots during dynamic locomotion. The proposed framework seamlessly combines deep learning, embedded computation, and real-time alerting to enhance the situational awareness of quadruped robots. Figure 6 illustrates the overall workflow of the proposed terrain identification system for a quadruped robot. The framework integrates offline deep learning model training with real-time embedded inference, enabling continuous terrain perception and timely situational awareness. During offline training, raw sensor data collected from the quadruped robot are first stored in CSV format and then processed by a feature preprocessing module. The sensor signals are normalized using Equation (1) to reduce scale variations and improve model stability. Subsequently, representative features are extracted from sensor measurements to capture terrain-related characteristics. A hyperparameter optimization process is applied to determine the optimal configuration of the TCN. Using the optimized parameters, the TCN model is trained to learn discriminative temporal patterns associated with different terrain types. The trained TCN model is then finalized for deployment and provides terrain identification outputs during inference. During the online deployment stage, the trained TCN model is deployed on an NVIDIA Jetson NX embedded platform to perform continuous inference on streaming sensor data from the quadruped robot. The embedded system continuously classifies the terrain in real time, enabling rapid environmental awareness. When specific terrain conditions or abnormal patterns are detected, the system automatically triggers a real-time email alert, providing timely notifications for monitoring and decision-making. Therefore, our proposed system effectively integrates deep learning, edge computing, and real-time communication to enable accurate and reliable terrain identification for quadruped robots operating in complex and unstructured environments. Table 2 presents the embedded system network architecture, including layers, interfaces, purposes, and configurations.

**Figure 7:**
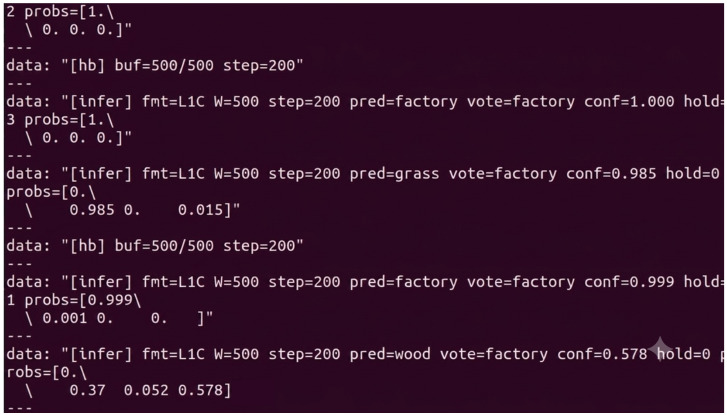
Real-time inference screen.

Figure 7 shows real-time terrain classification results executed on an NVIDIA Jetson Orin NX embedded platform. The system performs on-device inference using a sliding window and outputs predicted terrain classes with confidence scores, demonstrating stable and low-latency performance for real-time robotic sensing applications.

## 4. Experiential Results and Discussions

This section presents the experimental results of our proposed real-time terrain classification and slip detection system for quadruped robots, demonstrating its performance and reliability in dynamic environments. The proposed system was developed and evaluated on a workstation equipped with an NVIDIA RTX 2070 GPU (8 GB VRAM) and 32 GB DDR4 system memory. The data-processing pipeline and feature-extraction algorithms originally implemented in MATLAB R2023b were converted into optimized C source code using MATLAB Coder, enabling direct compilation and seamless execution on the embedded NVIDIA Jetson NX platform for real-time inference during online deployment. To ensure reliable communication and low-latency data exchange, a dual-network configuration was adopted: (1) Wlan0, dedicated to internal ROS communication within a private subnet (192.168.0.0/24), and (2) Wlan1, configured via DHCP/NAT for external connectivity, supporting real-time alerts and report transmission through SMTP/HTTP services. Moreover, the performance of the proposed real-time terrain classification system for quadruped robots was evaluated using confusion matrices, as well as accuracy, precision, and F1-score metrics, computed as follows [33,34]:

(10) $$\mathrm{Accuracy}=\frac{TP+TN}{TP+TN+FP+FN}$$

(11) $$\mathrm{Precision}=\frac{TP}{TP+FP}$$

where *TP*, *TN*, *FP*, and *FN* represent True Positive, True Negative, False Positive, and False Negative, respectively.

Furthermore, as shown in Table 3, we compare the performance of our proposed TCN model with other learning approaches, such as bagged tree, bidirectional long short-term memory (BiLSTM), 1D residual network (1D ResNet), and Conv1D+BiLSTM hybrid model, in terms of accuracy across various window sizes. As shown in the table, the proposed TCN model achieves superior accuracy across all window lengths, reaching 98.85% at a 5 s window. In comparison, the accuracies of BiLSTM, 1D ResNet, and Conv1D+BiLSTM at the same window are 96.30%, 98.44%, and 98.27%, respectively. The steady increase in accuracy with longer window sizes demonstrates that a wider temporal context enhances learning stability and feature representation. Therefore, based on the experimental results, the proposed TCN method exhibits better performance, robustness, and scalability for sequential terrain classification compared with other models presented in this study.

Moreover, we compare our proposed model with other deep learning techniques using precision, recall, and F1-score. Table 4 presents the comparison of models such as BiLSTM, 1DResNet, Conv1D+BiLSTM, and TCN. As shown by the results, TCN achieves the highest scores across all three metrics, with a precision of 0.989, a recall of 0.9884, and an F1-score of 0.9885, demonstrating its superior ability to classify terrain accurately. The other deep learning models, although performing well, show slightly lower values, indicating that TCN provides the most robust and consistent results among the considered architectures. This highlights the TCN model’s effectiveness at capturing temporal features for the given task.

**Figure 8:**
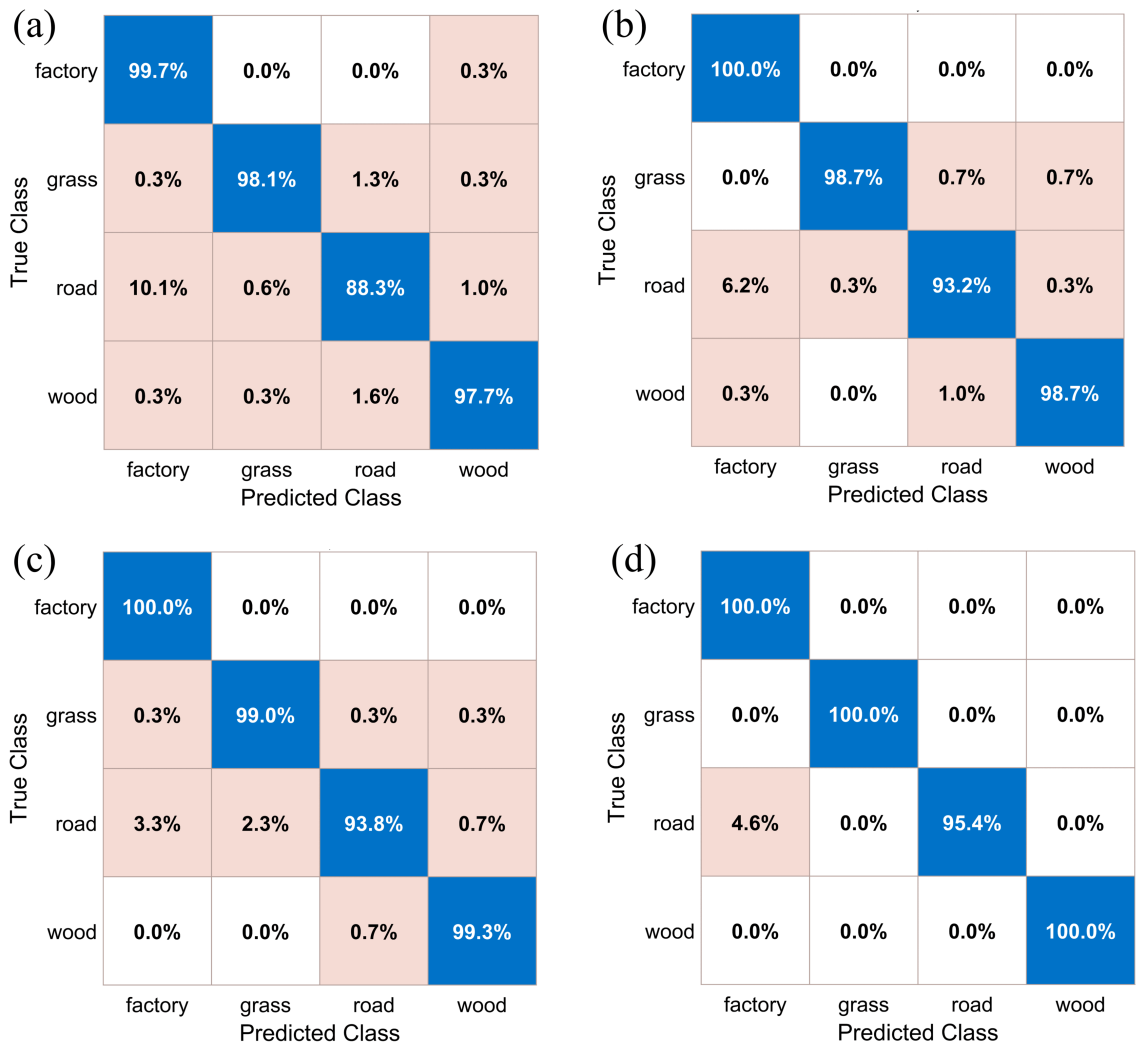
Confusion matrices of the proposed TCN model under different window lengths: (**a**) window lengths of 1 s, (**b**) window lengths of 2 s, (**c**) window lengths of 3 s, and (**d**) window lengths of 5 s.

Figure 8 shows the confusion matrices for the proposed TCN model with different window lengths, where the horizontal axis shows the predicted terrain classes and the vertical axis shows the actual terrain classes. As shown, the overall recognition performance improves as the window length increases. For window lengths of 1 s, 2 s, 3 s, and 5 s, the model achieves accuracies of 95.9%, 97.6%, 98.0%, and 98.8%, respectively. With shorter windows (1–2 s), slight confusion appears between similar terrains such as road and grass, due to limited temporal information. However, when the window length is extended beyond 3 s, the model captures more complete gait cycles, resulting in clearer diagonal dominance in the confusion matrices and fewer off-diagonal errors. This demonstrates that longer window lengths enable the TCN to extract richer temporal dependencies and more discriminative gait features, resulting in higher accuracy and more stable terrain recognition across all classes.

**Figure 9:**
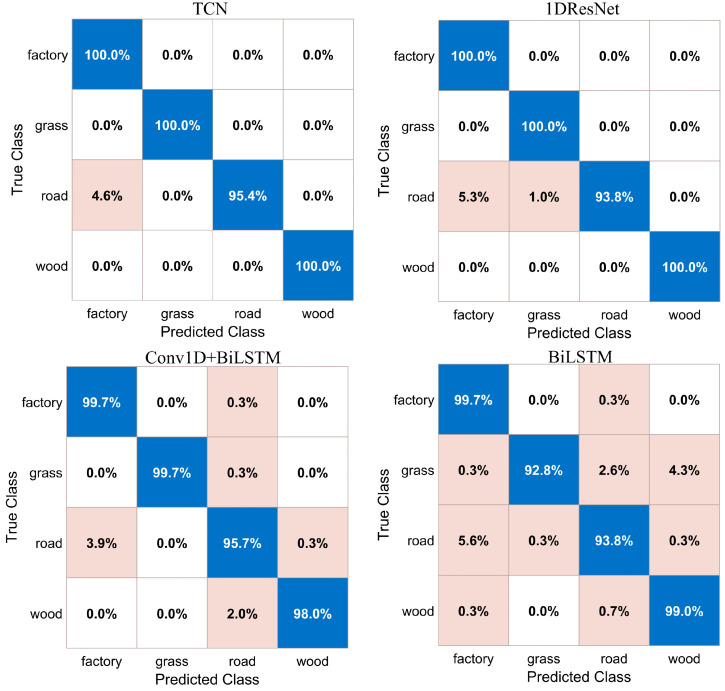
The confusion matrices for the proposed TCN, 1D ResNet, Conv1D+BiLSTM, and BiLSTM.

Moreover, Figure 9 shows the confusion matrices of the proposed model and other deep learning models such as 1D ResNet, Conv1D+BiLSTM, and BiLSTM. Among these models, the TCN model has the highest classification accuracy, predicting nearly perfect terrain classes (factory, grass, road, and wood) with minimal misclassification between road and factory. The performance of the 1D ResNet model has been reduced, especially for the road class. In addition, Conv1D+BiLSTM maintains solid accuracy overall but shows some confusion between road and wood surfaces. In contrast, the BiLSTM model in the figure exhibits the most noticeable misclassifications, especially for grass and roads. These results demonstrate the high precision and stability of the proposed TCN model when discriminating various terrain types.

**Figure 10:**
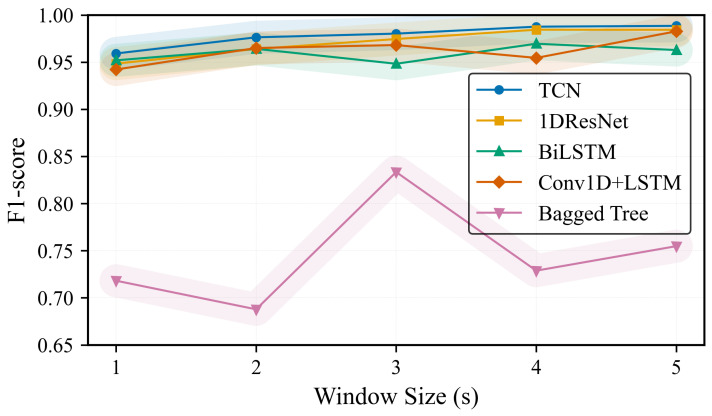
Comparison of models using F1-score under different window lengths (1–5 s).

Figure 10 shows the F1-score comparison across different window sizes for the proposed TCN model, 1DResNet, BiLSTM, Conv1D+LSTM, and the traditional Bagged Tree method. The proposed TCN consistently achieves the highest F1-scores, reaching 0.9885 at a window size of 5 s, while the Bagged Tree method reaches only 0.8334 at a window size of 3 s and 0.7548 at 5 s. This corresponds to an improvement of approximately 31% at the largest window size, highlighting the TCN’s superior ability to capture temporal dependencies and extract meaningful features from sequential data. Moreover, the TCN’s performance steadily increases with larger window sizes, whereas the Bagged Tree method remains unstable and significantly lower, demonstrating its limited capacity for modeling complex temporal patterns. These results clearly indicate that the proposed TCN significantly outperforms other methods and provides more reliable and accurate predictions for sequential signal classification.

**Figure 11:**
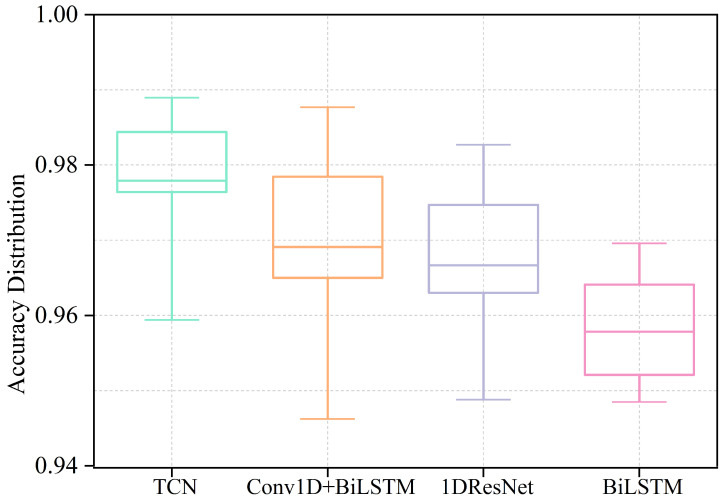
Comparison of model performance based on the distribution of accuracy.

Figure 11 presents the performance of different models in terms of accuracy distribution. The proposed TCN model exhibits the highest and most stable accuracy range among other models, demonstrating superior learning and generalization ability. The minimum, mean, and maximum accuracies of the proposed TCN model are 0.9594, 0.9778, and 0.9885, respectively, while those of Conv1D+BiLSTM, 1DResNet, and BiLSTM are (0.9423, 0.9695, 0.9877), (0.9488, 0.9667, 0.9827), and (0.9485, 0.9578, 0.9696), respectively. Furthermore, the proposed TCN model’s majority-accuracy distribution is highly concentrated, attaining the highest accuracy in the range 0.9764–0.9844. These results confirm that the TCN maintains consistently high accuracy across multiple window lengths with reduced performance fluctuation, indicating enhanced robustness and reliability in temporal feature extraction. Therefore, our proposed TCN-based system achieves a narrower and higher-accuracy range than other models, demonstrating superior stability and predictive precision in terrain classification for quadruped robots.

**Figure 12:**
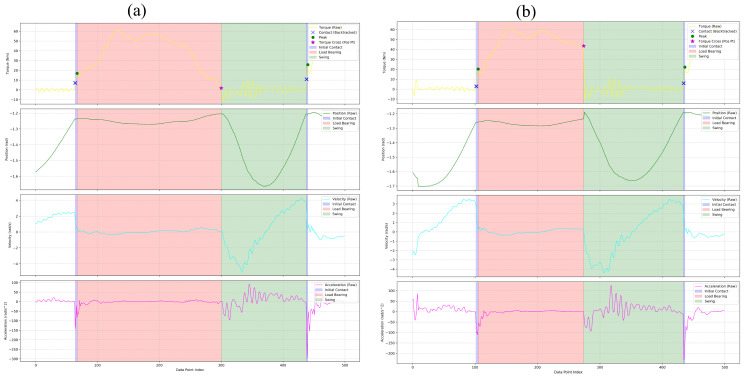
Slip dynamics during impact phase using KFE: (**a**) factory; (**b**) tile.

Moreover, this study presents a proprioceptive slip detection framework based on KFE joint dynamics and applies it to systematic terrain characterization under varying locomotion speeds and environmental conditions. Slip events are detected using a KFE-based impact-triggered algorithm that exploits the high sensitivity of the knee joint to ground contact dynamics. An impact is identified when the knee joint acceleration exceeds a predefined threshold in conjunction with a vertical foot velocity jump. Once triggered, a slip window is defined from the initial contact time $t_{\mathrm{start}}$ to the moment when the horizontal foot velocity reverses sign at $t_{\mathrm{end}}$. The sliding distance is quantified by integrating the horizontal foot velocity over this interval, as shown in Equation (9): Moreover, Figure 12 illustrates the dynamic response of the factory and tile surfaces during the impact phase. The temporal variations in torque, position, velocity, and acceleration are shown in the figure. In the figure, we present only two representative terrains, factory and tile. The shaded regions indicate the initial contact, load-bearing, and swing phases. The results show distinct differences in response characteristics between the two surfaces due to variations in friction coefficient. On the tile surface, the torque and acceleration signals exhibit larger fluctuations immediately after initial contact, indicating greater slip-induced instability. In contrast, the factory surface demonstrates smoother transitions with reduced oscillatory behavior, suggesting more stable ground interaction. The position and velocity profiles further confirm that the lower-friction tile surface introduces delayed stabilization and increased dynamic perturbations during load transfer. These observations validate the sensitivity of the proposed slip estimation framework in capturing terrain-dependent slip dynamics during the impact phase.

Table 5 summarizes slip performance on factory terrain under different locomotion speeds. Based on the experimental result analysis, fast locomotion increases the average slip distance by approximately 1.85×, from 0.011 to 0.020. The standard deviation also grows significantly, indicating less stable foot–ground contact at higher speeds. Maximum slip values rise slightly, highlighting the increased risk of instability during fast walking. Table 6 shows the impact of locomotion speed on slip distance in tile. Fast locomotion on tile terrain, a low-friction surface, significantly increases slip risk. The mean slip distance increases by approximately 1.75× (0.016 → 0.028 m), indicating a clear shift toward higher speed. The maximum slip reaches 0.2228 m, highlighting the extreme risk of dangerous slip events during high-speed walking. The standard deviation also increases, reflecting greater variability and less stable foot–ground contact. These results demonstrate that slip behavior is strongly influenced by both terrain properties and locomotion speed, particularly on low-friction surfaces. This observation highlights the necessity of accurate real-time terrain classification to enable speed-aware and terrain-adaptive locomotion control, thereby reducing slip risk and improving safety and stability for quadruped robots operating in real-world environments.

**Figure 13:**
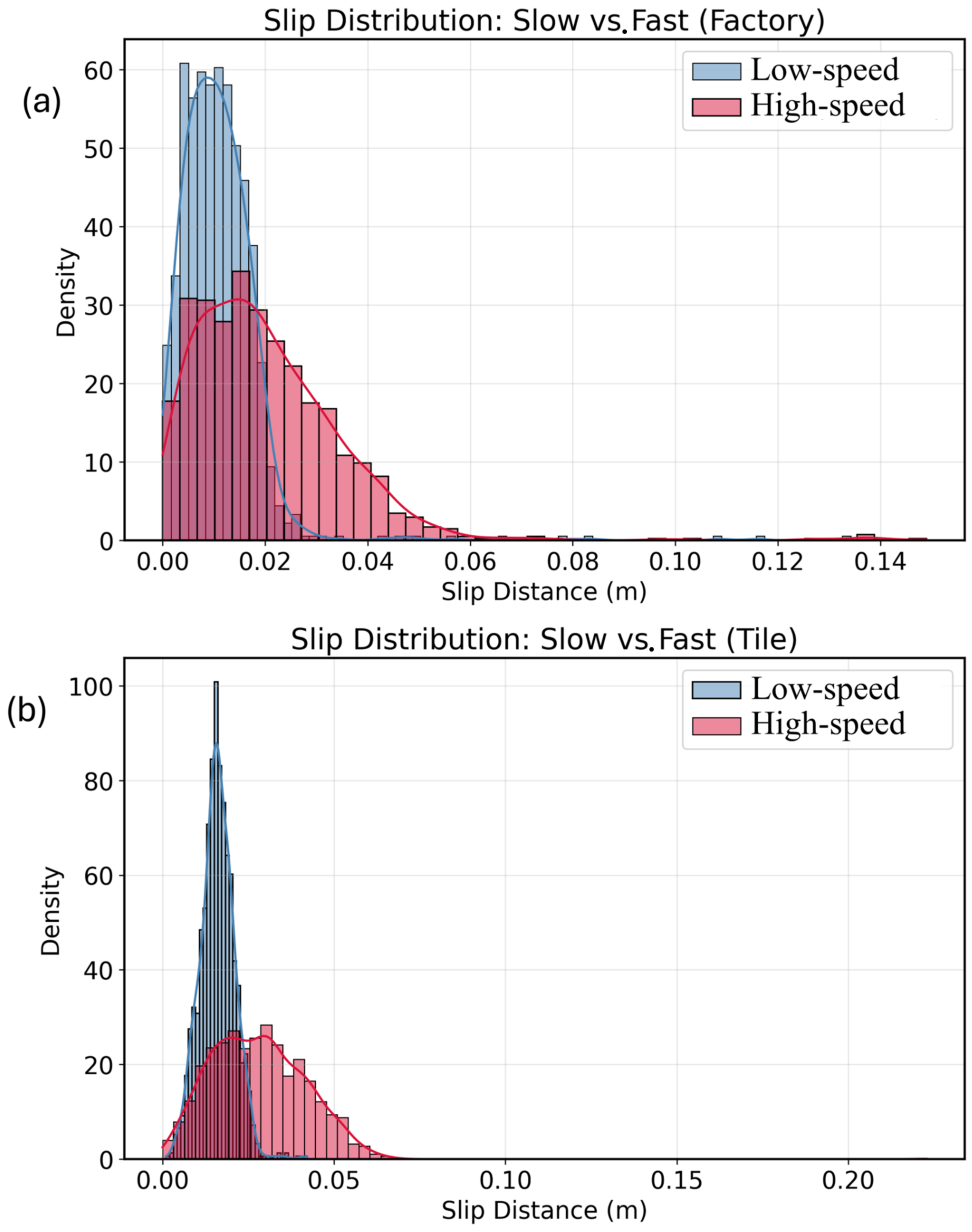
Slip distance distribution under slow (low speed) and fast (high speed) locomotion speeds: (**a**) factory; (**b**) tile.

The experimental results in Table 7 demonstrate that terrain material, environmental conditions, and locomotion speed all significantly influence slip behavior. Among the three tested surfaces, tile exhibits the highest slip risk, whereas road (interlocking bricks) provides excellent traction under dry conditions. Environmental factors further amplify slip on interlocking bricks; the mean slip doubles when wet compared to dry conditions, even exceeding the slow-speed slip observed on factory floors, highlighting the critical role of surface moisture. The highest average slipping distance is 0.0279 m on tile at fast speed and the lowest is 0.0031 m on dry road/brick at fast speed. Finally, high-speed locomotion significantly increases slip risk on both tile and factory surfaces, underscoring the need for speed-aware, terrain-adaptive control strategies in quadruped robots. Figure 13 shows the effect of speed on slip distance in the factory and tile terrain. Moreover, the proposed method was compared with previous studies based solely on slip mitigation performance using the average slipping distance. The average slipping distance was defined as the displacement between the intended foothold position and the actual contact point during locomotion, where a smaller value indicates better slip suppression. Yan et al. [35] reported an average slipping distance of $6.33\times 10^{-3}\;m$, whereas the proposed method reduced this value to $3.10\times 10^{-3}\;m$, corresponding to an improvement of approximately 51%. This result demonstrates that the proposed real-time slip estimation method provides more precise slip detection and significantly improves slip mitigation.

**Figure 14:**
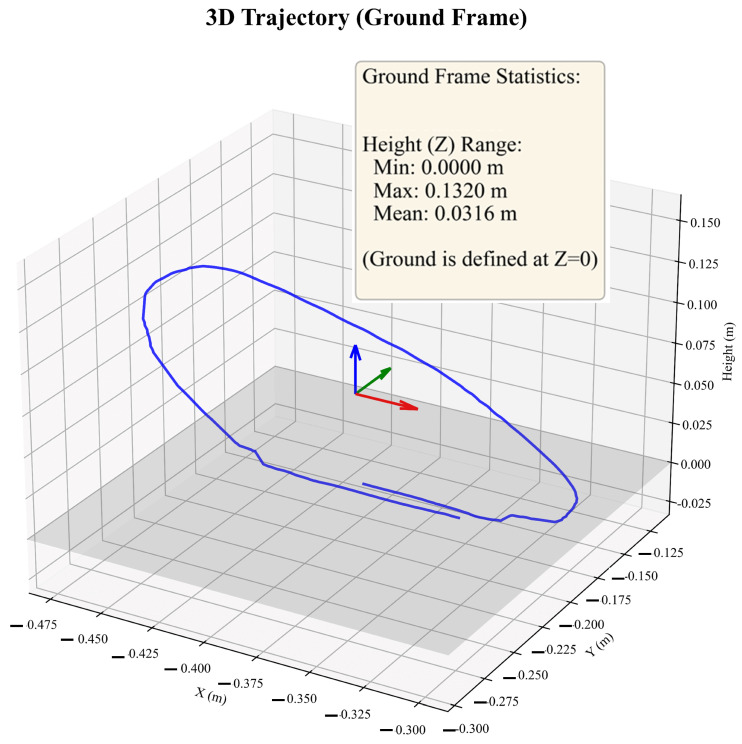
Reconstructed 3D foot-end trajectory of the right hind (RH) leg in the ground frame.

Figure 14 illustrates the reconstructed 3D foot-end trajectory in the ground coordinate frame, obtained via forward kinematics from experimentally measured joint states. The blue curve represents the continuous spatial trajectory relative to the ground plane (defined at Z = 0), while the shaded plane denotes the reference ground surface. The red, green, and blue arrows indicate the X, Y, and Z axes of the ground frame, respectively. The figure helps to visually relate the reconstructed leg trajectory to the observed robot motion and to make the slip-related movement easier to identify. The reconstructed trajectory exhibits a vertical height range from 0 to 0.132 m, with a mean elevation of 0.0316 m. Near-zero height segments are interpreted as the stance phase, during which the foot remains close to the ground plane, whereas elevated portions correspond to the swing phase of the leg. The values are reported as descriptive statistics of the reconstructed trajectory and are used to support the qualitative interpretation of the stance–swing motion pattern.

Table 8 shows that the proposed method was experimentally validated on a real ANYmal D quadruped robot and achieved an accuracy of 98.8%, outperforming previous studies. In addition, the proposed method provides quantitative slip-distance estimation under real-world terrain conditions, achieving the lowest measured average slipping distance of $3.10\times 10^{-3}$ m. Compared with Yan et al. [36], the proposed method exhibits a lower average slipping distance under the tested conditions. Therefore, the proposed method not only improves terrain-recognition accuracy but also provides a quantitative basis for evaluating slip severity.

## 5. Conclusions

In this work, we presented a novel real-time terrain classification framework for quadruped robots based on proprioceptive sensors and temporal convolutional networks (TCNs). The proposed system relies entirely on proprioceptive signals. Thus, it overcomes limitations related to lighting variations, occlusions, and high cost. By transforming joint torque data into the frequency domain, the system effectively captures the dynamic interaction between the robot’s legs and the terrain. The TCN model further enhances performance by learning temporal dependencies within the torque spectrum, enabling accurate and robust terrain classification. Experimental validation on a real quadruped robot demonstrates that the proposed method achieves 98.8% detection accuracy, outperforming traditional sensor-dependent and feature-based approaches. The proposed TCN method consistently outperforms the traditional Bagged Tree across all window sizes, achieving F1-score improvements of approximately 33.6%, 41.9%, 17.6%, 35.5%, and 30.9% for window sizes 1 s, 2 s, 3 s, 4 s, and 5 s, respectively, highlighting its robust ability to model temporal dependencies in sequential data.

Furthermore, the proposed system was validated through extensive real-world experiments on a quadruped robot and deployed on a Jetson Orin NX embedded edge computing platform using ONNX runtime, achieving real-time inference with a latency of less than 0.1 s at a sensing frequency of 100 Hz. The results confirm that the system operates efficiently in real time and adapts well to dynamic and unstructured environments. Moreover, real-time experimental analysis demonstrates that terrain material, environmental conditions, and locomotion speed significantly influence slip behavior, highlighting the importance of terrain-aware and speed-adaptive strategies for safe quadruped locomotion. Thus, the proposed approach provides a cost-effective, reliable, and scalable solution, offering a significant step toward greater autonomy, adaptability, and environmental awareness in quadruped robotic systems.

## Figures and Tables

**Table 1:** Hyperparameters of the proposed TCN model.

Parameter	Value
Batch size	128
Optimizer	Adam
Activation function	ReLU
Learning rate	$1\times 10^{-4}$
Dropout rate	0.5
Loss function	Cross entropy

**Table 2:** Embedded System Network Architecture.

Layer	Interface	Purpose/Configuration
Application	ROS Nodes	Real-time data
Network	Wlan0	Real-time ROS: 192.168.0.0/24
Network	Wlan1	External I/O: DHCP/NAT

**Table 3:** Comparison of models in terms of accuracy with various window lengths.

Accuracy in Various Window Lengths (1–5 s)					
Model	1 s	2 s	3 s	4 s	5 s
Bagged Tree	0.7665	0.7778	0.8103	0.7391	0.6875
BiLSTM	0.9521	0.9641	0.9485	0.9696	0.9630
1DResNet	0.9488	0.9641	0.9747	0.9844	0.9844
Conv1D+BiLSTM	0.9423	0.9650	0.9681	0.9549	0.9827
**TCNN**	**0.9594**	**0.9764**	**0.9804**	**0.9877**	**0.9885**

**Table 4:** Performance of different deep learning models using precision, recall, and F1-score.

Model	Precision	Recall	F1-Score
BiLSTM	0.9639	0.9630	0.9628
1DResNet	0.9851	0.9844	0.9843
Conv1D+BiLSTM	0.9829	0.9827	0.9827
**TCN**	**0.9890**	**0.9884**	**0.9885**

**Table 5:** Impact of locomotion speed on slip distance (factory).

Speed	Mean Slip (m)	Std Dev (m)	Max Slip (m)
Slow	0.0110	0.0091	0.1342
Fast	0.0204	0.0158	0.1490

**Table 6:** Impact of locomotion speed on slip distance (tile).

Speed	Mean Slip (m)	Std Dev (m)	Max Slip (m)
Slow	0.0160	0.0050	0.0421
Fast	0.0279	0.0140	0.2228

**Table 7:** Comparison of average slip distance across three terrains under varying speeds and environmental conditions.

Terrain and Condition	Mean Slip (m)	Slip Risk Level
Road (Brick) Slow (Wet)	0.0066	Low
Factory Slow	0.0110	Low
Tile Slow	0.0160	Medium
Road (Brick) Fast (Dry)	0.0031	Very Low
Factory Fast	0.0204	Medium
Tile Fast	0.0279	High

**Table 8:** Comparison of the proposed method with previous studies in quadruped robots.

Author and References	Robot/Platform	Validation Type	Accuracy (%)	Average Slipping Distance (m)
Sun Yan et al. [35]	Unitree A1	Real	97.89	$6.33\times 10^{-3}$
Yan et al. [36]	Webots	Simulation	93.19	Not reported
This work	ANYmal D	Real	98.8	$3.10\times 10^{-3}$

## Data Availability

The data presented in this study are available in this article.
